# Targeting Antibiotics to Households for Trachoma Control

**DOI:** 10.1371/journal.pntd.0000862

**Published:** 2010-11-02

**Authors:** Isobel M. Blake, Matthew J. Burton, Anthony W. Solomon, Sheila K. West, María-Gloria Basáñez, Manoj Gambhir, Robin L. Bailey, David C. W. Mabey, Nicholas C. Grassly

**Affiliations:** 1 Department of Infectious Disease Epidemiology, Imperial College London, London, United Kingdom; 2 Department of Infectious and Tropical Diseases, London School of Hygiene and Tropical Medicine, London, United Kingdom; 3 Dana Center for Preventive Ophthalmology, Wilmer Eye Institute, Johns Hopkins University, Baltimore, Maryland, United States of America; 4 MRC Centre for Outbreak Analysis and Modelling, Imperial College London, London, United Kingdom; University of California San Francisco, United States of America

## Abstract

**Background:**

Mass drug administration (MDA) is part of the current trachoma control strategy, but it can be costly and results in many uninfected individuals receiving treatment. Here we explore whether alternative, targeted approaches are effective antibiotic-sparing strategies.

**Methodology/Principal Findings:**

We analysed data on the prevalence of ocular infection with *Chlamydia trachomatis* and of active trachoma disease among 4,436 individuals from two communities in The Gambia (West Africa) and two communities in Tanzania (East Africa). An age- and household-structured mathematical model of transmission was fitted to these data using maximum likelihood. The presence of active inflammatory disease as a marker of infection in a household was, in general, significantly more sensitive (between 79% [95%CI: 60%–92%] and 86% [71%–95%] across the four communities) than as a marker of infection in an individual (24% [16%–33%]–66% [56%–76%]). Model simulations, under the best fit models for each community, showed that targeting treatment to households has the potential to be as effective as and significantly more cost-effective than mass treatment when antibiotics are not donated. The cost (2007US$) per incident infection averted ranged from 1.5 to 3.1 for MDA, from 1.0 to 1.7 for household-targeted treatment assuming equivalent coverage, and from 0.4 to 1.7 if household visits increased treatment coverage to 100% in selected households. Assuming antibiotics were donated, MDA was predicted to be more cost-effective unless opportunity costs incurred by individuals collecting antibiotics were included or household visits improved treatment uptake. Limiting MDA to children was not as effective in reducing infection as the other aforementioned distribution strategies.

**Conclusions/Significance:**

Our model suggests that targeting antibiotics to households with active trachoma has the potential to be a cost-effective trachoma control measure, but further work is required to assess if costs can be reduced and to what extent the approach can increase the treatment coverage of infected individuals compared to MDA in different settings.

## Author Summary

Repeated ocular infection with the bacterium *Chlamydia trachomatis* leads to the development of trachoma, a major cause of infectious blindness worldwide. Mass distribution of antibiotics, a component of the current trachoma control strategy, has had success in reducing infection in some areas, but results in a large number of uninfected people receiving antibiotics. We have previously shown that transmission of the bacteria between people in the same household is very efficient. Here, we investigated the effectiveness and cost-effectiveness of targeting antibiotics to households with active trachoma (inflammatory disease) compared to mass distribution, using data from four trachoma-endemic populations and a mathematical model of transmission. We found a high correspondence between households with active trachoma and infected households. In all populations the household targeted approach was predicted to be as effective as mass distribution, but it reduced the number of uninfected individuals receiving antibiotics, making the targeted strategy more cost-effective when antibiotics are not donated. Assuming antibiotics are donated, we predicted the targeted strategy to be more cost effective if it increases the proportion of infected individuals receiving treatment. Further work to address the feasibility and the cost variability in implementing the targeted approach in different settings is now required.

## Introduction

Trachoma, a ‘Neglected Tropical Disease’, is the leading infectious cause of blindness worldwide and there are currently an estimated 46 million people with the active stage of the disease [1]. The disease mostly affects impoverished populations where people cannot afford treatment and access to running water is scarce.

The World Health Organization (WHO) advocates the ‘SAFE’ strategy (Surgery for trichiasis, distribution of Antibiotics, Facial cleanliness and Environmental improvement) to work towards the Global Elimination of Trachoma as a public health problem by 2020. Annual mass drug administration (MDA) of antibiotics, to reduce the prevalence of the aetiological bacterium, *Chlamydia trachomatis*, is recommended for at least three years to members of communities in which the prevalence of Trachomatous Inflammation – Follicular (TF) in 1–9 year-olds is 10% or greater [2]. WHO recommends azithromycin as the first-line (oral) antibiotic for all, except infants under the age of 6 months who are given topical tetracycline [2]. MDA is advocated because screening individuals is not cost-effective and there is a poor correlation between active disease and infection of an individual [3], [4], [5], [6]. Field-ready and cost-effective diagnostic tests for infection with *C. trachomatis* are currently unavailable.

The ‘SAFE’ strategy has had success in reducing the prevalence of active trachoma in certain populations [7], [8], [9]. However, there are many costs associated with implementing MDA, particularly if the antibiotics are not donated, and many uninfected individuals receive treatment [10], [11], [12]. It has been estimated that there are fifty-seven countries endemic for trachoma [13]. Control programmes in eighteen of these countries currently receive azithromycin donated by the manufacturer, Pfizer, through the International Trachoma Initiative [14]. However a large disparity remains in certain countries between the number of individuals in the target population requiring treatment and the number of individuals receiving antibiotics [14].

If antibiotics can be successfully targeted to groups of infected individuals within a population rather than being administered to the whole population, the number of antibiotic doses required per population may be reduced. This saving of antibiotic resources could be utilised by other populations who require treatment to reduce trachoma. However the targeting method would only be justified if the method is as effective in reducing transmission as MDA and is also cost-effective.

Households with active trachoma are potential targets for antibiotic distribution. Trachoma clusters by household [15], [16], [17], [18] and we have previously shown that in most communities intra-household transmission is very efficient [19]. We found on average 71% of incident infections to be the result of household transmission (with the remainder due to transmission between households). An alternative approach to targeting treatment would be to limit treatment to children because they are the principal reservoir and source of infection in most communities. Children in some communities have been shown to have a relatively high prevalence of active disease [20], [21], [22] and a high burden of infection [23].

Here we investigate whether targeting antibiotics to households that have at least one member with active disease or to children alone is effective in the prevention of ocular chlamydial infection by analysing data on the prevalence of *C. trachomatis* and active disease from four endemic populations in West and East Africa (two in The Gambia, and two in Tanzania) with different baseline trachoma prevalence. We calculate the cost-effectiveness of targeted household treatment compared with MDA on the basis of a mathematical model and previously published data on the costs of these interventions [10], [11].

## Methods

### Data Collection and Ethical Considerations

Conjunctival swabs were collected from a total of 4,436 individuals living in four endemic populations, which had not received prior interventions for trachoma control, in West and East Africa (Upper Saloum District and Jali village in The Gambia; Kahe Mpya sub-village and Maindi village in Tanzania) and the presence of infection was assessed using Polymerase Chain Reaction (PCR) amplification of a target sequence in the common cryptic plasmid of the bacterium *C. trachomatis*. Standard procedures current at the time of these surveys were followed to prevent contamination, described in [23] and [24]. In Maindi village, quantitative PCR amplification of the *omp1* gene was used to indicate presence of infection. In all four studies clinical observations were made by experienced trained observers using a ×2.5 binocular loupe and pen torch or direct sunlight. In The Gambia the more detailed clinical diagnosis “FPC” system [25] was used but subsequently converted to the simplified WHO grading system [26] for this analysis. In Tanzania the simplified grading system was used. Active disease was defined as the presence of TF and / or Trachomatous Inflammation – Intense (TI). Detailed demographic information was collected including individual age, gender, and household membership. Full descriptions of the study populations and laboratory methods have been published elsewhere [17], [24], [27], [28] and details on community structure are summarized in [19]. Pre-control prevalences of infection in these populations (all ages) were 7.2%, 22.1%, 9.5% and 36.0% respectively. The age distribution of the prevalence of infection in these four communities is given in Table S1. The proportion of people present and consented to being screened for trachoma in the four data sets was 0.84 Upper Saloum district, 0.98 Kahe Mpya sub-village, 0.99 Jali village, and 0.86 for Maindi village. The work presented in this paper is based on further analyses of the data obtained in the original studies which had been granted ethical clearance [17], [24], [27], [28] and did not involve collecting further information. For this reason additional ethical approval was not sought.

### Sensitivity and Specificity of Active Disease as Marker of Infection

The sensitivity and specificity of active disease (TF and TI) as a marker of infection were calculated among individuals in each community. We also calculated the sensitivity and specificity of active disease exhibited by at least one member of a household as a marker for infection of at least one household member (which we refer to as the household sensitivity and specificity).

### Model of Infection Transmission

Ocular chlamydial infection probably elicits only a limited protective immune response against re-infection and can be described by a simple Markov model where each individual may be either susceptible or infected. We have previously analyzed a susceptible→infected→susceptible (SIS) model where the population is structured into households [19], [29], [30]. Here we have extended this model to allow for different transmission parameters among ‘children’ (those aged less than ten years) and ‘adults’ (those individuals aged 10 years and older) (Text S1 ‘*Model of Ocular Chlamydia Transmission*’). We chose this classification of age because children under the age of ten are considered to be the principal reservoir of infection. Transmission parameters of each model for each dataset were estimated from the survey data using maximum likelihood, assuming endemic equilibrium. The most parsimonious yet adequate model for each dataset was selected using the Akaike Information Criterion (AIC) [31]. The transmission model was written in R (version 2.7.2). The rate of recovery from infection was taken as the reciprocal of the average duration of infection estimated from a Gambian cohort with frequent follow-up [4] (18.6 weeks for children, 7.1 weeks for adults and 17.2 weeks on average for the non-age-structured model).

### Stochastic Simulations of Treatment Scenarios

The effectiveness of different treatment strategies was assessed using the most parsimonious model identified for each of the communities (Text S1 ‘*Model Selection*’, Table S2). With the exception of Upper Saloum district, the transmission models included a greater contribution of children to transmission than adults. Active disease at the household level was incorporated into the model at each round of treatment. At the time of treatment, each household was assigned a disease status by sampling from a Bernoulli distribution where the probability of a household having at least one individual with active disease was taken to be a function of the number of infected individuals within a household at the time of treatment. This probability function was calculated for each dataset on the basis of the observed distribution of infection and active disease in households of different sizes (Figure S1).

The outcome of three annual rounds of azithromycin treatment was investigated in all four populations as this is the number of treatment rounds recommended by the WHO prior to re-assessment of the prevalence of active disease when the baseline prevalence of TF in children is greater than 10%. For a transmission model parameterised to Maindi village, annual rounds were predicted to result in infection returning to almost baseline level in all strategies within one year after a treatment round suggesting that the treatment rounds need to be more frequent for this higher transmission setting. Therefore the effect of six bi-annual rounds was investigated for this setting.

Stochastic simulations of the model were used to examine four possible treatment scenarios:

- MDA, in which the aim is to treat everyone in the community but a certain proportion of individuals is missed;
- Household targeted treatment (HTT) of households with one or more members presenting with active disease in a household but a proportion of individuals is missed;
- HTT of households with one or more member presenting with active disease and all members within such household are treated;
- MDA of children aged <10 years only, assuming that a certain proportion is missed.

A single treatment with azithromycin was assumed to be 95% efficacious in clearing infection [32]. We did not explicitly model treatment of infants aged <6 months with topical tetracycline instead of oral antibiotics. We assumed treatment coverage to be 80% in a), b) and d). One hundred simulations were run for each strategy to compare the effectiveness of each strategy. Further details of the stochastic model (written in R) are given in Text S1 *‘Stochastic Simulation Model’*.

### Cost-Effectiveness

The cost-effectiveness of different antibiotic distribution strategies (compared with the ‘doing nothing’ option) from a government and societal perspective was assessed using previously published cost data from Mali and Nepal [10], [11] (summarised in Table S3) and the results from the stochastic simulations. The cost data were collected in 1998 and 2000 respectively for the studies in Mali and Nepal. Using the most recently available consumer price index for the two countries (2007) [33], [34], [35], the costs were converted to the value of US$ in 2007. Costs included the generic price of azithromycin per tablet, drug delivery costs per population size, and opportunity costs (the amount of money not earned per recipient whilst they attend the treatment campaign). Delivery costs in the Mali study consisted of governmental (salaries and vehicle investment) and distribution (dispatching, training of nurses and other health workers, per diems and fuel) costs specific to each strategy. Delivery costs in the study from Nepal were composed of salary and transportation costs and not the training of health workers. The delivery costs were higher for household-targeted treatment as they accounted for the extra training and salaries of nurses to diagnose trachoma in Mali and the increase in transport costs in Nepal (in this study two trips per community were assumed for this strategy: one for screening and one for treatment). We assumed that MDA was distributed via a central site. In agreement with the study in Mali, we assumed that opportunity costs equal to half a day or one hour's wages were incurred by individuals aged ≥10 years receiving treatment during MDA or HTT respectively. We assumed that individuals aged 10 years or older received an average of 3.43 azithromycin tablets and those under the age of 10 received an average of 1.02 tablets. (Text S1 *‘Cost Effectiveness Analysis’*). One hundred stochastic simulations were performed for each strategy in each community and the costs were applied to the resulting simulations. The total cost of azithromycin was calculated by multiplying the number of individuals receiving treatment by the price per tablet and the mean number of tablets received in that age group. The delivery costs were scaled linearly to the size of the population in the four endemic areas under study and were assumed to occur at each round of treatment.

A discount rate of 3% per year was applied to all costs. Two estimates of total drug costs, delivery costs and opportunity costs were obtained using the two different sets of cost data and the mean cost of the two was calculated. Cost-effectiveness was calculated on the basis of the median effectiveness observed in the simulations, with lower and upper bounds based on the inter-quartile range of the simulations and the upper and lower costs from the two cost studies.

## Results

### Active Disease as a Marker of Infection

In all four communities (Upper Saloum district and Jali village in The Gambia, and Kahe Mpya sub-village and Maindi village in Tanzania) the sensitivity of active disease as a marker of infection was higher and specificity lower at the household level compared with the individual level (Table 1). Limiting clinical diagnosis in a household to children under the age of 10 years resulted in a similar household sensitivity and specificity compared to undertaking clinical diagnosis in all age groups (Table S4).

**Table 1:** Sensitivity and specificity of active trachoma as a marker of infection.

Population	Sensitivity		Specificity	
	Individual level	Household level	Individual level	Household level
Upper Saloum District, The Gambia	0.24 [0.16–0.33]	0.79 * [0.60–0.92]	0.93 [0.92–0.96]	0.64 * [0.53–0.74]
Jali village, The Gambia	0.63 [0.57–0.70]	0.86 * [0.71–0.95]	0.95 [0.94–1]	0.77 * [0.46–0.95]
Kahe Mpya sub-village, Tanzania	0.66 [0.56–0.76]	0.80 [0.68–0.90]	0.84 [0.82–0.87]	0.58 * [0.49–0.66]
Maindi village, Tanzania	0.63 [0.57–0.69]	0.84 * [0.77–0.90]	0.74 [0.70–0.78]	0.58 * [0.47–0.68]

Results are shown for four trachoma endemic communities in West and East Africa (Upper Saloum and Jali in The Gambia, and Kahe-Mpya and Maindi in Tanzania). Numbers in square brackets indicate 95% binomial confidence intervals. The symbol * indicates statistical significance (p<0.05) between the individual and the household level using Fisher's exact test.

### Stochastic Simulations

**Figure 1:**
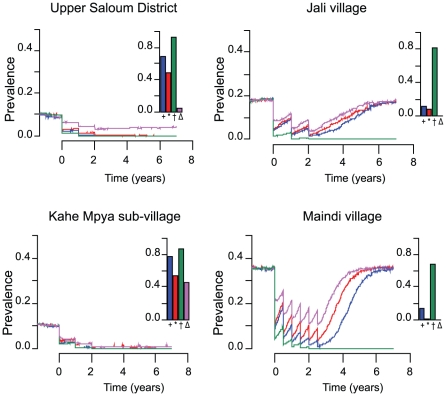
Comparison of MDA with HTT or MDA of children <10 years old only. The y-axis represents prevalence of ocular *C. trachomatis* infection. Blue (+) – MDA with 80% coverage, Red (*) – HTT with 80% coverage, Green (†) – HTT with 100% coverage, Purple (Δ) – MDA of children under the age of 10 years old with 80% coverage. Treatment rounds commence at time = 0. Upper Saloum district, Kahe Mpya sub-village and Jali village have three annual treatment rounds, Maindi village has six biannual rounds because of the high baseline prevalence of infection. 100 stochastic simulations were run for each scenario and the median of these simulations at each time point are displayed here. The bar charts show the probability of eliminating infection from the community for each treatment scenario. MDA = Mass drug administration. HTT = Household targeted treatment.

Targeting treatment to households, in which at least one resident has active disease, was predicted to result in post-treatment dynamics similar to MDA (Figure 1). The household-targeted approach had a slightly higher rate of return of infection and therefore the probability of eliminating infection five years after the last treatment round was predicted to be somewhat lower than the probability of eliminating infection after MDA (absolute difference between the probabilities in each setting was −0.22, −0.04, −0.25 and −0.12 for Upper Saloum district, Jali village, Kahe Mpya sub-village and Maindi village respectively). However if all individuals in targeted households were treated, then the probability of eliminating infection was predicted to greatly increase in each setting, being greater than MDA (absolute difference between the probability of eliminating infection after HTT with 100% coverage within the targeted households and the probability of eliminating infection after MDA was 0.26, 0.69, 0.07 and 0.44 respectively) (Figure 1). Limiting MDA to children under the age of 10 years resulted in an initial decrease in the prevalence of infection in the untreated older population (Figure S2) but the probability of eliminating infection in the whole community was greatly reduced compared to the other treatment scenarios investigated (Figure 1). There was a relatively smaller difference in effectiveness between the different treatment scenarios in the communities with relatively low baseline prevalence (Upper Saloum district and Kahe-Mpya sub-village) but HTT with 100% coverage, remained the most effective treatment scenario.

Modifying the model to account for variation in the efficiency of transmission among households resulted in faster return of infection for all treatment strategies in the simulations and the probability of eliminating infection was lower five years after the last treatment round (Figure S3). However, the relative impact of the different strategies remained robust to this additional complexity.

### Cost-Effectiveness

**Figure 2:**
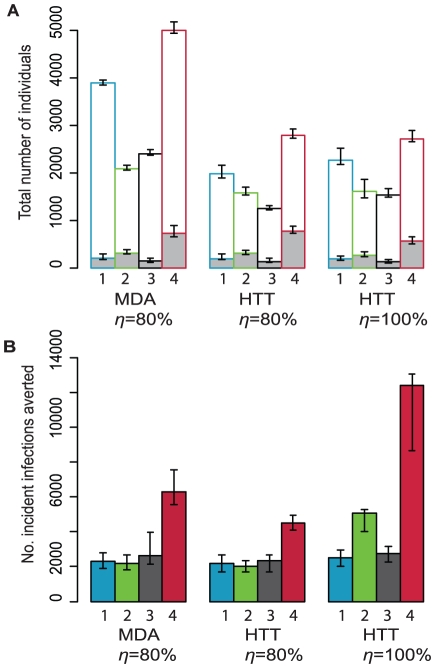
Total number of individuals receiving antibiotics and incident infections averted for MDA compared with HTT. Coloured bars correspond to the different communities: Blue (1) – Upper Saloum District, Green (2) – Jali village, Black (3) – Kahe Mpya sub-village and Red (4) – Maindi village. A) The grey bars correspond to the total number of infected individuals receiving antibiotics and the white bars correspond to the number of uninfected individuals receiving antibiotics. B) The total number of incident infections averted is from the start of treatment through to 5 years after the last round of treatment. For both panels Upper Saloum district, Kahe Mpya sub-village and Jali village have three annual treatment rounds, Maindi village has six biannual rounds. 100 stochastic simulations were run for each scenario and the median of these simulations at each time point are displayed here. The error bars correspond to the inter-quartile range. Therapeutic coverage is donated by *η*. MDA = Mass drug administration. HTT = Household targeted treatment.

A household-targeted approach resulted in a similar number of infected individuals receiving treatment compared with MDA, but reduced the number of treatments given to uninfected individuals (Figure 2A). Assuming 80% therapeutic coverage and that azithromycin was not donated, HTT was predicted to be more cost-effective than MDA in all four communities when including the cost of generic azithromycin (Table 2). Assuming azithromycin was donated, HTT was predicted to be more cost effective when opportunity costs for individuals collecting drugs in the MDA approach were included (Table 2). Otherwise, MDA was estimated to be more cost effective. We did not calculate the cost-effectiveness of targeting treatment to children because the model simulations showed it to be the least effective of the four treatment scenarios at controlling infection.

**Table 2:** Cost-Effectiveness (CE) of Azithromycin MDA compared to HTT.

		Upper Saloum district			Jali village			Kahe Mpya sub-village			Maindi village		
Strategy		MDA	HTT	HTT	MDA	HTT	HTT	MDA	HTT	HTT	MDA	HTT	HTT
Therapeutic Coverage (%)		80	80	100	80	80	100	80	80	100	80	80	100
Total cost of azithromycin		6,988 [4,630–9,408]	3,420 [2,080–4,937]	3,963 [2,403–5,675]	3,781 [2,488–5,094]	2,764 [1,707–3,952]	2,677 [1,565–4,064]	4,315 [2,846–5,827]	2,180 [1,357–3,076]	2,683 [1,677–3,781]	9,010 [5,950–12,143]	4,920 [3,075–6,882]	4,778 [2,984–6,800]
Delivery Cost		288 [222–353]	386 [296–476]	386 [296–476]	152 [118–187]	205 [157–252]	205 [157–252]	176 [136–217]	237 [181–292]	237 [181–292]	364 [281–447]	488 [374–602]	488 [374–602]
Opportunity Cost (OC)		1030 [730–1336]	125 [82–175]	146 [95–201]	563 [397–732]	102 [67–141]	99 [62–145]	638 [451–831]	80 [53–109]	99 [75–134]	1344 [950–1745]	181 [122–244]	177 [118–243]
CE (2007 US$ per infection averted) excluding OC	Incl. AZT cost	3.1 [1.8–5.0]	1.7 [1.0–2.7]	1.7 [1.0–2.6]	1.8 [1.0–2.8]	1.5 [0.9–2.1]	0.6 [0.4–0.8]	1.7 [1.0–2.7]	1.0 [0.6–1.8]	1.0 [0.7–1.6]	1.5 [0.8–2.2]	1.2 [0.8–1.6]	0.4 [0.3–0.8]
	Excl. AZT cost	0.12 [0.08–0.18]	0.18 [0.11–0.28]	0.15 [0.10–0.24]	0.07 [0.04–0.10]	0.10 [0.07–0.15]	0.04 [0.03–0.06]	0.06 [0.05–0.10]	0.10 [0.07–0.17]	0.09 [0.06–0.13]	0.06 [0.04–0.08]	0.11 [0.08–0.15]	0.04 [0.03–0.07]
CE (2007 US$ per infection averted) including OC	Incl. AZT cost	3.6 [2.0–5.7]	1.8 [1.0–2.8]	1.8 [1.1–2.7]	2.1 [1.2–3.2]	1.5 [0.9–2.2]	0.6 [0.4–0.9]	1.9 [1.2–3.1]	1.1 [0.7–1.8]	1.1 [0.7–1.6]	1.7 [1.0–2.5]	1.2 [0.8–1.7]	0.4 [0.3–0.8]
	Excl. AZT cost	0.6 [0.3–0.9]	0.2 [0.1–0.4]	0.2 [0.1–0.3]	0.3 [0.2–0.4]	0.2 [0.1–0.2]	0.1 [0.0–0.1]	0.3 [0.2–0.5]	0.1 [0.1–0.2]	0.1 [0.1–0.2]	0.3 [0.2–0.4]	0.2 [0.1–0.2]	0.1 [0.0–0.1]

Costs are in 2007 US$. Costs are given as the average of the total costs estimated from the two different cost studies [10], [11] multiplied by the respective median from 100 simulations with the number in square brackets giving the lower and upper bounds (see Text S1). Upper Saloum district, Kahe Mpya sub-village and Jali village have three annual treatment rounds; Maindi village has six biannual rounds. Cost effectiveness was calculated as the cost per incident infection averted from the start of treatment through to five years after the last treatment round. AZT = azithromycin. OC = opportunity cost. HTT = Household Targeted Treatment.

If a visit to a household facilitates treatment of all members, then there was a large increase in the number of incident infections averted compared with either MDA or targeted approaches with 80% coverage in hyperendemic settings (Figure 2B). As a result, the household targeting strategy in which all members of diseased households are treated was predicted to be significantly more cost-effective in the areas with high baseline prevalence, even when azithromycin was assumed to be donated (Table 2).

## Discussion

A targeted approach for distributing azithromycin would result in fewer antibiotic doses distributed per head of population than MDA, thus saving medication for use by other trachoma endemic populations in need of treatment to reach ‘the Global Elimination of Trachoma as a public health problem by the year 2020’. However the approach would only be warranted if it is as effective in reducing ocular *C. trachomatis* prevalence in a population as MDA and as cost-effective.

Our results have indicated that targeting antibiotics to households with at least one member with active disease has a similar effect to MDA in the reduction of infection. Active disease was found not to be 100% sensitive as a marker of infection at the household level and this explains the small differences observed between the two strategies. However, we have shown that HTT results in a large reduction in the number of uninfected individuals receiving antibiotics compared to MDA (26%–51% reduction). When antibiotics were assumed to be donated, opportunity costs incurred by individuals taking time to collect tablets from the MDA program resulted in HTT being more cost-effective. Although the large majority of trachoma control programmes currently operate using donated azithromycin, we also estimated the cost-effectiveness of HTT assuming antibiotics were purchased at the generic price, to give a monetary value to the amount of antibiotic used in each strategy for the donor's perspective of the strategy and because some small scale programmes operating at village levels do purchase the drug [36], [37]. In this case the dominating cost was that of the antibiotics and so HTT was estimated to be more cost-effective.

If all members of visited households were assumed to be treated as a result of the visit by the treatment team, a much higher chance of eliminating infection from the community in all settings compared with MDA was predicted. The success of this approach will depend on the extent of household transmission and the degree to which household visits can boost treatment coverage. For example, in a community such as Kahe Mpya where household transmission was estimated to be limited [19], this approach can be hypothesised to be less effective. Baseline surveys of the prevalence of disease could be used as an indicator for the likely degree of household transmission, enabling the selection of communities that would benefit from a targeted approach. A large effort is typically required to achieve high coverage levels for MDA control programs [38]. In contrast, analogy can be drawn with other disease control programmes, such as vaccination for polio and measles, in which a house-to-house strategy of administering vaccination achieves much higher coverage than a fixed point campaign [39], [40]. Whether all household members can be reached with a single household visit remains to be investigated and further work is required to address whether coverage of infected individuals can be improved with HTT at what additional costs.

The cost per incident infection averted was greatly reduced when 100% of targeted-household members were assumed to be treated in areas with a relatively high prevalence of infection at baseline (Jali and Maindi villages), both when assuming azithromycin was and was not donated. In low prevalence settings the additional benefits of treating all household members were less apparent in our simulations because we investigated the effect of only three annual rounds of treatment, which, in these settings, were sufficient for any treatment scenario to have a greater than 50% chance at eliminating infection.

There are some caveats to our cost analysis: the cost data used in the study are a decade old and the linear scaling of delivery costs to the size of each community may not be appropriate for some costs (for example the time taken to perform a round of HTT may depend not only on the size but also on the geography of the population). However, the two cost studies referred to were the only published cost data at the time of our study that included the full cost of HTT. We assumed that individuals aged ≥10yr received a mean of 3.4 tablets whilst those aged<years received 1 tablet. This is a simplification and does not include azithromycin suspension given to younger children and topical tetracycline given to infants under 6 months. However, this would increase the total cost of antibiotics further, making targeted treatment more cost-effective.

We assumed that MDA occurred via a central site distribution. The WHO states that MDA can be carried out either via central site or by house to house distribution [2]. If we had assumed the latter for MDA there would have been a smaller difference in the distribution costs between MDA and HTT, (the only difference would be the cost of screening for active disease) and so HTT would have appeared more cost-effective in comparison to MDA. We took the assumption from the Mali cost study that MDA via a central site would result in adult antibiotic recipients having an opportunity cost of half a day's wages and HTT one hour's wages. However the WHO advises that MDA should be performed outside of the farming season [2] to try to minimise opportunity costs and improve the treatment coverage. In our analysis opportunity costs had a small impact on the cost-effective estimates but further studies could be performed to analyse what proportion of the recipient population's activities are interrupted by the different treatment campaigns.

The costs involved in treatment scenarios are likely to vary from country to country and by size of the community treated. Our work has investigated HTT in populations of approximately 1,000 people. If such an approach were to be implemented on a district or even country-wide scale, economies of scale will have to be considered *e.g.* a large number of nurses (or volunteers) will have to be trained for screening and there may be societal costs incurred as such personnel may stop working on other health programmes. Further studies are required to investigate these differences.

The delivery costs of targeting treatment to diseased households could be reduced in a number of ways which need to be researched further. We currently assume separate visits to households to assess disease and provide antibiotics. Assessment and treatment could be administered in a single visit, thereby reducing transport and salary costs. Furthermore, village volunteers could be trained to assess clinical disease to reduce the costs of ophthalmic nurses (a scheme which has been trialled with success in Ghana [41]). We also assumed that all residents would be screened for active disease at each round of HTT. Firstly, this could be limited to children under the age of ten: we have shown here that this approach has the same sensitivity as screening all ages but the difference in cost between the two approaches remains to be ascertained. Secondly, in practice, as soon as one person in a household is found to have active trachoma, the remainder of the household would not need to be screened. Therefore the cost of HTT in this work may be an overestimate in the higher prevalence settings where it is likely that in some households not all residents would be required to be screened. Further data are required to elucidate how the cost of screening for identifying target households will vary for different levels of prevalence and household clustering, including settings where WHO currently recommends HTT (active trachoma prevalence of 5%–9% in 1–9 year olds).

Data on active trachoma, analysed in this study, were collected in a scientific setting by experienced observers. The accuracy of trachoma grading may be more variable in a programmatic setting. A consequence of this would be that that sensitivity and specificity of active disease as a marker of infection at the individual level could worsen. However, this may be less significant at the household level, where diagnosis of just a single case of active disease is sufficient for treatment of that household. Further field studies would help understand the implications of trachoma grading error on HTT.

The original analyses of the cost data from Nepal and Mali differed from our work. The study in Nepal [11], [42] compared MDA of children to HTT of all ages. The study found the two strategies not to be significantly different from one another in the reduction of active disease and the costs involved (although this could be explained in part by the low power of the study). The original study in Mali [10] found HTT to be significantly less effective than MDA of the whole population with respect to the reduction of active disease prevalence one year after one round of treatment (although the age-adjusted odds ratio for prevalence active disease after HTT in relation to MDA was 1.56 with 95% confidence intervals of 1.00–2.43 indicating the strategies could have had the same outcome). The study found HTT to be more cost-effective except in low transmission settings. A difference between our work and the previous cost analyses is that here the cost was calculated as a cost per incident infection averted over five years rather than a change in point prevalence between baseline and one time point in the previous cost analysis. Measuring the number of infections avoided is not feasible in the field but measuring the cost-effectiveness in this way from model simulations gives a better insight into the impact of each treatment scenario on cumulative exposure to infection and therefore the ocular disease process.

Limiting treatment to children is another way to target treatment. Our models predicted that the prevalence in adults declines when children under the age of ten are treated, in agreement with House *et al.* [43], but this strategy is not as effective as MDA or HTT because the probability of eliminating infection is reduced in all four communities. Women could be included along with children in the target group as explored in the study in Mali [10]. However we did not investigate this strategy because the number of transmission parameters to be estimated would have been too large for the size of our dataset and the prevalence of infection did not differ largely between males and females in the study communities (excluding Maindi) [23]. Besides, there is considerable risk that specifically excluding adult males from treatment schedules would jeopardise community support for drug distribution.

Another method to target treatment would be to ‘graduate’ communities from MDA once the prevalence of ocular *C. trachomatis* infection is below a certain threshold, as suggested by Ray and colleagues [44]. Their study predicted graduating communities to be efficacious and drug-sparing (assuming a diagnostic test for infection becomes available in a field-ready format), by fitting a stochastic model allowing for heterogeneous transmission between communities, to the Upper Saloum district and Kahe Mpya sub-village data and a group of communities in Ethiopia.

Therefore two separate analyses of the Tanzanian and Gambian data sets have resulted in two different suggestions for targeting treatment. Here we fitted and simulated under a model of transmission which allows individuals to be infected by an infected member of their household or community at two different rates, specific to the setting. The Upper Saloum district contains 14 villages and this analysis grouped the villages together as one population. The Tanzanian sub-village contains balozis (groups of roughly 10 households that form an administrative unit) that we also grouped together. Additional analysis would be required to understand the relationship between within household transmission and heterogeneous community transmission where several of the communities constitute a larger population. This would then allow comparisons of the different targeting strategies to be made.

A recent study in Ethiopia [45] found that communities which had received MDA with azithromycin was associated with an odds ratio of 0.51 (0.29–0.90) for childhood (1–9 years) mortality one year after commencement of MDA compared to children in communities which did not receive the antibiotic. If this phenomenon extends to other settings then the impact of HTT with azithromycin on child mortality should be examined.

Caveats to our model of transmission have been described previously [19]. Infection status of individuals was characterised through PCR of ocular swabs. Standard precautions at the time of data collection were performed to prevent contamination of infection data (although the risk of contamination cannot fully be ruled out due to the absence of negative field controls). Sensitivity analysis of the assumption that each household is at equal risk of becoming infected found that increasing the level of heterogeneity in the household transmission parameters resulted in a faster rate of return of infection after treatment with a lower probability of eliminating infection for each treatment strategy. Further studies are needed to quantify differences in households' risk of becoming infected. Individuals were assumed not to move from one age group to the next but this is a reasonable simplification as the time spent in the lower age group (ten years) by each individual is far longer than the average duration of infection. We have assumed that the relationship between active disease and infection remains constant in a household after treatment. This requires further investigation but preliminary analyses of follow-up data from Upper Saloum District and Kahe Mpya sub-village indicates that households with at least one person with active disease at baseline can predict which households will contain individuals with ocular chlamydial infection at follow-up time points more accurately than households with active disease at follow-up.

The model did not include interventions to improve facial cleanliness (F) or the environment (E), the interventions advocated by WHO to accompany the distribution of antibiotics [2]. The exclusion of these interventions allowed the predicted effectiveness and cost-effectiveness of the different distribution strategies to be shown clearly. Inclusion of ‘F’ and ‘E’ would reduce the rate of return of infection and increase the probability of eliminating infection by an uncertain factor but is unlikely to alter the rank order of the impact of the different distribution strategies. If the cost of implementing ‘F’ and ‘E’ is independent of the antibiotic distribution strategy then the relative differences between the cost-effectiveness of implementing trachoma control for different antibiotic distribution strategies would remain unchanged. The exclusion of ‘F’ and ‘E’ from the model may explain why infection was observed to return relatively slowly in Maindi village following two rounds of treatment whereas our model predicts infection to rapidly return for an area with such a high baseline prevalence of infection. Changes in hygiene could have arisen in the village through residents receiving radio broadcasts by the National Trachoma Control Programme informing individuals to improve face washing and latrine usage [46] or alternatively, by simply the presence of the intervention itself, altering individuals' behaviour.

Our model suggests that targeting treatment to households that have at least one resident with active trachoma is as effective as MDA in a diverse variety of settings and can be more effective if the strategy increases the coverage of infected individuals. We also show that HTT is drug-sparing and has the potential to be more cost-effective but to have a better understanding of this in settings for which azithromycin is donated, more studies are required to evaluate whether HTT can improve antibiotic coverage levels of infected individuals and whether the cost can be further reduced compared with costs recorded in the studies in Mali and Nepal. The results of these studies will provide a better understanding of efficient and effective antibiotic distribution approaches for trachoma control programmes in countries with limited resources.

## Supporting Information

Figure S1 The probability of a household having one or more members with active disease, given a certain number of infected individuals, calculated for the four endemic populations.
(0.59 MB EPS)

Figure S2 Reduction in prevalence of ocular C. trachomatis infection in adults and children when only children <10 years receive MDA. The transmission parameters used were those estimated by fitting the model to the data from the respective communities. The lines are median values of 100 stochastic simulations. The black line corresponds to prevalence in ‘children’ <10 years and the grey line corresponds to ‘adults’, aged ≥10 years old.
(0.66 MB EPS)

Figure S3 Role of heterogeneity in household transmission parameters for controlling infection. Blue - Mass treatment with 80% coverage, Red - Treatment targeted at households with one or more individuals with active disease but 80% of individuals in each household receive treatment, Green - Treatment targeted at households with one or more individuals with active disease and 100% of individuals in each household are treated, Purple - MDA of children under the age of 10 years old with 80% coverage. Treatment rounds occur at times = 0, 1 and 2. The model fitted to Jali village was used as an example as it has reasonably high baseline prevalence and a large amount of household transmission. The bar chart shows the probability of eliminating infection after three rounds of household targeted treatment for communities with varying levels of heterogeneity of household susceptibility. Slight heterogeneity corresponds to an overdispersion parameter of 5 and strong heterogeneity corresponds to an overdispersion parameter of 1.5.
(0.89 MB EPS)

Table S1 The number of individuals and prevalence of infection for each age group in four trachoma endemic communities. The numbers in the square brackets are 95% binomial confidence intervals.
(0.03 MB DOC)

Table S2 Comparison of Different Trachoma Transmission Models Fitted to Infection Data.
(0.04 MB DOC)

Table S3 Cost Data from Mali and Nepal summarised from [10] and [11].
(0.03 MB DOC)

Table S4 Sensitivity (a) and specificity (b) of active disease as a marker of infection in the household (both limiting clinical diagnosis to children under 10 years old and assessing clinical disease in all ages) for four trachoma endemic communities. Numbers in square brackets indicate 95% binomial confidence intervals. There is no statistical significant difference between assessing active disease in children under ten years old and assessing disease in all ages (Fisher's exact test p>0.05).
(0.03 MB DOC)

Table S5 Maximum likelihood estimates of the transmission parameters for each of the four populations and nested models. The numbers in square brackets denote 95% confidence intervals.
(0.51 MB DOC)

Text S1 Extra information of the methods: Model of Ocular Chlamydia transmission, Parameter Estimation, Model selection, Stochastic simulation and cost-effectiveness analysis.
(0.42 MB DOC)
