# Supplementary material for: Targeting Antibiotics to Households for Trachoma Control
Source: PLoS Negl Trop Dis. 2010 Nov 2;4(11):e862. doi: 10.1371/journal.pntd.0000862 (PMC2970531; doi:10.1371/journal.pntd.0000862)
Supplement: Table S1 — The number of individuals and prevalence of infection for each age group in four trachoma endemic communities. The numbers in the square brackets are 95% binomial confidence intervals. (0.03 MB DOC) [file pntd.0000862.s004.doc]

**Table S1. The number of individuals and prevalence of infection for each age group in four trachoma endemic communities.** The numbers in the square brackets are 95% binomial confidence intervals

| **Population** | **Individuals <10 years old** | | **Individuals ≥ 10 years old** | |
| --- | --- | --- | --- | --- |
| Number of villages, District, and Country | Number of individuals | Prevalence of infection [95% CI] | Number of individuals | Prevalence of infection [95% CI] |
| 14 villages, Upper Saloum District, The Gambia | 600 | 0.09 [0.07 – 0.11] | 995 | 0.06 [0.04 – 0.07] |
| Jali village, Kiang West District, The Gambia | 304 | 0.40 [0.34 – 0.46] | 542 | 0.11 [0.08 – 0.14] |
| Sub-village of Kahe Mpya, Rombo District, Tanzania | 362 | 0.16 [0.12 – 0.20] | 616 | 0.06 [0.04 – 0.08] |
| Maindi village, Kongwa District, Tanzania | 363 | 0.47 [0.41 – 0.53] | 654 | 0.29 [0.25 – 0.33] |
