# Supplementary material for: Targeting Antibiotics to Households for Trachoma Control
Source: PLoS Negl Trop Dis. 2010 Nov 2;4(11):e862. doi: 10.1371/journal.pntd.0000862 (PMC2970531; doi:10.1371/journal.pntd.0000862)
Supplement: Table S2 — Comparison of Different Trachoma Transmission Models Fitted to Infection Data. (0.04 MB DOC) [file pntd.0000862.s005.doc]

| Model | Number of transmission parameters estimated | Description | AIC | | | |
| --- | --- | --- | --- | --- | --- | --- |
|  |  |  | The Gambia | | Tanzania | |
|  |  |  | Upper Saloum district | Jali village | Kahe Mpya sub-village | Maindi village |
| 1 | 1 | Homogeneous mixing of the population. Children and adults recover at the same rate. | 462 | 398 | 445 | 753 |
| 2 | 2 | Household and community transmission. Children and adults recover at the same rate. | 305* | 361 | 422 | 714 |
| 3 | 2 | Household and community transmission. Children recover more slowly than adults. | 301 | 293 | *395* * | 695* |
| 4 | 3 | Household and community transmission. Adults are *θ* times as infectious as children. Children recover more slowly than adults. | 302 | 292 | 397 | *693* |
| 5 | 3 | Household and community transmission. Adults are *θ* times as susceptible as children. Children recover more slowly than adults. | *300* | *279* * | 396 | 695 |
| 6 | 4 | Household and community transmission. Adults have a different infectiousness to that of children and this may differ between household and community transmission. Children recover more slowly than adults. | 304 | 294 | 399 | 695 |
| 7 | 4 | Household and community transmission. Adults have a different susceptibility from that of children and this may differ between household and community transmission. Children recover more slowly than adults. | 302 | 280 | 398 | 694 |
| 8 | 6 | Household and community transmission. Adults and children are equally susceptible and infectious but there are different contact rates between adults→ adults, adults→ children, children → adults and children→ children. Children recover more slowly than adults. | 306 | 285 | 402 | 694 |
| 9 | 8 | The transmission rates of an infected individual infecting a susceptible individual of age classes: adults→ adults, adults→ children, children→ adults and children→ children differ for both household and community transmission. Children recover more slowly than adults. | 307 | 284 | 405 | 697 |

**Table S2 Comparison of Different Trachoma Transmission Models Fitted to Infection Data**

The Akaike Information Criterion (AIC) values are shown for each model and each of the four endemic communities under investigation. The smallest AIC value for each community is indicated in italics. If there is a difference greater than or equal to 10 between two AIC values there is enough empirical support for the model with the lower AIC value (4). The model with enough empirical support is indicated by an asterisk (*).
