# Supplementary material for: Targeting Antibiotics to Households for Trachoma Control
Source: PLoS Negl Trop Dis. 2010 Nov 2;4(11):e862. doi: 10.1371/journal.pntd.0000862 (PMC2970531; doi:10.1371/journal.pntd.0000862)
Supplement: Table S3 — Cost Data from Mali and Nepal summarised from [10] and [11]. (0.03 MB DOC) [file pntd.0000862.s006.doc]

**Table S3 Cost Data from Mali and Nepal summarised from [10] and [11**].

| 1. **Mali** | | |
| --- | --- | --- |
|  | Cost (US$) in 1998 | Adjusted to 2007 US$ |
| Generic cost per tablet | 0.43 | 0.50 |
| Government cost for mass treatment per community of 510,000 people | 1,000 | 1,160 |
| Government cost for household-targeted treatment per community of 510,000 people | 10,000 | 11,600 |
| Distribution cost for mass treatment per community of 510,000 people | 20,000 | 23,200 |
| Distribution cost for targeted treatment per community of 510,000 people | 18,000 | 20,880 |
| Opportunity cost per adult per day | 0.55 | 0.64 |

| 1. **Nepal** | | |
| --- | --- | --- |
|  | Cost (US$) in 2000 | Adjusted to 2007 US$ |
| Generic cost per tablet | 0.64 | 0.99 |
| Total distribution and governmental cost for mass treatment per child | 0.049 | 0.08 |
| Total distribution and governmental cost for targeted treatment per child | 0.066 | 0.10 |
| Adult wage per hour | 0.09 | 0.14 |
