# Supplementary material for: Targeting Antibiotics to Households for Trachoma Control
Source: PLoS Negl Trop Dis. 2010 Nov 2;4(11):e862. doi: 10.1371/journal.pntd.0000862 (PMC2970531; doi:10.1371/journal.pntd.0000862)
Supplement: Table S4 — Sensitivity (a) and specificity (b) of active disease as a marker of infection in the household (both limiting clinical diagnosis to children under 10 years old and assessing clinical disease in all ages) for four trachoma endemic communities. Numbers in square brackets indicate 95% binomial confidence intervals. There is no statistical significant difference between assessing active disease in children under ten years old and assessing disease in all ages (Fisher's exact test p>0.05). (0.03 MB DOC) [file pntd.0000862.s007.doc]

**Table S4** . Sensitivity (a) and specificity (b) of active disease as a marker of infection in the household (both limiting clinical diagnosis to children under 10 years old and assessing clinical disease in all ages) for four trachoma endemic communities. Numbers in square brackets indicate 95% binomial confidence intervals. There is no statistical significant difference between assessing active disease in children under ten years old and assessing disease in all ages (Fisher’s exact test p>0.05).

| **Population** | **Sensitivity** | | **Specificity** | |
| --- | --- | --- | --- | --- |
|  | Assessing all ages | Assessing children only | Assessing all ages | Assessing children only |
| Upper Saloum District, The Gambia | 0.79 [0.60 – 0.92] | 0.79 [0.60 – 0.92] | 0.64 [0.53 – 0.74] | 0.69 [0.58 – 0.78] |
| Jali village, The Gambia | 0.86 [0.71 – 0.95] | 0.86 [0.71 – 0.95] | 0.77 [0.46 – 0.95] | 0.77 [0.46 – 0.95] |
| Kahe Mpya sub-village | 0.80 [0.68 – 0.90] | 0.79 [0.65 – 0.88] | 0.58 [0.49 – 0.66] | 0.65 [0.57 – 0.74] |
| Maindi village | 0.84 [0.77 – 0.90] | 0.78 [0.70 – 0.84] | 0.58 [0.47 – 0.68] | 0.64 [0.53 – 0.74] |
