# Supplementary material for: Targeting Antibiotics to Households for Trachoma Control
Source: PLoS Negl Trop Dis. 2010 Nov 2;4(11):e862. doi: 10.1371/journal.pntd.0000862 (PMC2970531; doi:10.1371/journal.pntd.0000862)
Supplement: Table S5 — Maximum likelihood estimates of the transmission parameters for each of the four populations and nested models. The numbers in square brackets denote 95% confidence intervals. (0.51 MB DOC) [file pntd.0000862.s008.doc]

**Table S5 Maximum likelihood estimates of the transmission parameters for each of the four populations and nested models. The numbers in square brackets denote 95% confidence intervals**

| 1. **Upper Saloum district, The Gambia** | | | |  |  |  |  |  |  |  |
| --- | --- | --- | --- | --- | --- | --- | --- | --- | --- | --- |
| **Model number** | $\beta_{Lcc}$ | $\beta_{Lca}$ | $\beta_{Laa}$ | $\beta_{Lac}$ | $\beta_{Gcc}$ | $\beta_{Gca}$ | $\beta_{Gaa}$ | $\beta_{Gac}$ | $\theta$ | $\ln L$ |
| 1 | NA | NA | NA | NA | 1.62 [1.60 - 1.65] | $\beta_{Gcc}$ | $\beta_{Gcc}$ | $\beta_{Gcc}$ | NA | -229.95 |
| 2 | 1.78 [1.71 - 1.86] | $\beta_{Lcc}$ | $\beta_{Lcc}$ | $\beta_{Lcc}$ | 0.20 [0.17 - 0.23] | $\beta_{Gcc}$ | $\beta_{Gcc}$ | $\beta_{Gcc}$ | NA | -150.67 |
| 3 | 2.52 [2.44 - 2.60] | $\beta_{Lcc}$ | $\beta_{Lcc}$ | $\beta_{Lcc}$ | 0.26 [0.23 - 0.30] | $\beta_{Gcc}$ | $\beta_{Gcc}$ | $\beta_{Gcc}$ | NA | -148.70 |
| 4 | 0.37 [0.34 - 0.40] | $\beta_{Lcc}$ | $\beta_{Lcc}*\theta$ | $\beta_{Lcc}*\theta$ | 0.04 [0.03 - 0.04] | $\beta_{Gcc}$ | $\beta_{Gcc}*\theta$ | $\beta_{Gcc}*\theta$ | 20.88 [19.62 - 22.22] | -147.85 |
| 5 | 2.13 [1.77 - 2.56] | $\beta_{Lcc}*\theta$ | $\beta_{Lcc}*\theta$ | $\beta_{Lcc}$ | 0.24 [0.15 - 0.37] | $\beta_{Gcc}*\theta$ | $\beta_{Gcc}*\theta$ | $\beta_{Gcc}$ | 1.48 [1.40 - 1.56] | -147.23 |
| 6 | 0.37 [0.35 - 0.40] | $\beta_{Lcc}$ | 7.74 [6.64 - 9.02] | $\beta_{Laa}$ | 0.41 [0.37 - 0.44] | $\beta_{Gcc}$ | 0.01 [0.00 – 5x10^17^] | $\beta_{Gaa}$ | NA | -147.85 |
| 7 | 1.96 [1.56 - 2.46] | $\beta_{Laa}$ | 3.35 [2.52 - 4.46] | $\beta_{Lcc}$ | 0.35 [0.34 - 0.36] | $\beta_{Gaa}$ | 0.21 [0.17 - 0.26] | $\beta_{Gcc}$ | NA | -146.86 |
| 8 | 1.88 [0.78 - 4.51] | 2.42 [1.02 - 5.72] | 4.44 [1.86 - 10.61] | $\beta_{Lca}$ | 0.45 [0.19 - 1.08] | 0.05 [0.00 - 8.3x10^2^] | 0.55 [0.27 - 1.10] | $\beta_{Gca}$ | NA | -147.10 |
| 9 | 3.04 [2.69 - 3.45] | 3.72 [1.68 - 8.21] | 3.25 [0.90 -11.79 ] | 0.09 [0.00 – 4x10] | 0.54 [0.49 - 0.59] | 0.16 [0.00 - 13.15] | 0.18 [0.00 - 69.46] | 0.29 [0.08 - 0.98] | NA | -145.69 |

| 1. **Jali village, The Gambia** | | | |  |  |  |  |  |  |  |
| --- | --- | --- | --- | --- | --- | --- | --- | --- | --- | --- |
| **Model number** | $\beta_{Lcc}$ | $\beta_{Lca}$ | $\beta_{Laa}$ | $\beta_{Lac}$ | $\beta_{Gcc}$ | $\beta_{Gca}$ | $\beta_{Gaa}$ | $\beta_{Gac}$ | $\theta$ | $\ln L$ |
| 1 | NA | NA | NA | NA | 1.94 [1.89 - 2.01] | $\beta_{Gcc}$ | $\beta_{Gcc}$ | $\beta_{Gcc}$ | NA | -198.10 |
| 2 | 1.63 [1.29 - 2.05] | $\beta_{Lcc}$ | $\beta_{Lcc}$ | $\beta_{Lcc}$ | 0.45 [0.25 - 0.79] | $\beta_{Gcc}$ | $\beta_{Gcc}$ | $\beta_{Gcc}$ | NA | -178.34 |
| 3 | 2.35 [1.85 - 2.98] | $\beta_{Lcc}$ | $\beta_{Lcc}$ | $\beta_{Lcc}$ | 0.65 [0.37 - 1.13] | $\beta_{Gcc}$ | $\beta_{Gcc}$ | $\beta_{Gcc}$ | NA | -144.33 |
| 4 | 0.56 [0.03 - 9.04] | $\beta_{Lcc}$ | $\beta_{Lcc}*\theta$ | $\beta_{Lcc}*\theta$ | 0.11 [0.01 - 1.59] | $\beta_{Gcc}$ | $\beta_{Gcc}*\theta$ | $\beta_{Gcc}*\theta$ | 12.48 [0.47 - 334.06] | -143.06 |
| 5 | 3.26 [2.47 - 4.31] | $\beta_{Lcc}*\theta$ | $\beta_{Lcc}*\theta$ | $\beta_{Lcc}$ | 0.76 [0.04 - 1.37] | $\beta_{Gcc}*\theta$ | $\beta_{Gcc}*\theta$ | $\beta_{Gcc}$ | 0.47 [0.32 - 0.69] | -136.37 |
| 6 | 0.56 [0.04 - 8.71] | $\beta_{Lcc}$ | 6.95 [4.07 - 11.87] | $\beta_{Laa}$ | 0.71 [0.00 - 5e+5] | $\beta_{Gcc}$ | 0.09 [0.00 – 2x10^88^] | $\beta_{Gaa}$ | NA | -143.06 |
| 7 | 3.07 [2.09 - 4.50] | $\beta_{Laa}$ | 1.65 [1.12 - 2.44] | $\beta_{Lcc}$ | 0.89 [0.04 - 1.99] | $\beta_{Gaa}$ | 0.26 [0.06 - 1.15] | $\beta_{Gcc}$ | NA | -136.23 |
| 8 | 3.29 [2.09 - 5.17] | 1.77 [0.74 - 4.21] | 1.40 [0.00 - 50.52] | $\beta_{Lca}$ | 1.24 [0.59 - 2.57] | 0.10 [0.00 - 282.90] | 0.78 [0.02 - 33.78] | $\beta_{Gca}$ | NA | -136.40 |
| 9 | 0.53 [0.44 - 0.62] | 0.48 [0.46 - 0.51] | 4.37 [3.01 - 6.35] | 17.17 [11.78 - 25.03] | 0.04 [0.00 - 209.75] | 0.59 [0.50 - 0.69] | 0.11 [0.00 - 13.19] | 2.52 [2.48 - 2.55] | NA | -134.16 |

| 1. **Kahe Mpya sub-village, Tanzania** | | | |  |  |  |  |  |  |  |
| --- | --- | --- | --- | --- | --- | --- | --- | --- | --- | --- |
| **Model number** | $\beta_{Lcc}$ | $\beta_{Lca}$ | $\beta_{Laa}$ | $\beta_{Lac}$ | $\beta_{Gcc}$ | $\beta_{Gca}$ | $\beta_{Gaa}$ | $\beta_{Gac}$ | $\theta$ | $\ln L$ |
| 1 | NA | NA | NA | NA | 1.67 [1.64 - 1.70] | $\beta_{Gcc}$ | $\beta_{Gcc}$ | $\beta_{Gcc}$ | NA | -221.67 |
| 2 | 0.95 [0.62 - 1.45] | $\beta_{Lcc}$ | $\beta_{Lcc}$ | $\beta_{Lcc}$ | 0.90 [0.67 - 1.22] | $\beta_{Gcc}$ | $\beta_{Gcc}$ | $\beta_{Gcc}$ | NA | -208.91 |
| 3 | 1.41 [1.16 - 1.71] | $\beta_{Lcc}$ | $\beta_{Lcc}$ | $\beta_{Lcc}$ | 1.20 [1.04 - 1.37] | $\beta_{Gcc}$ | $\beta_{Gcc}$ | $\beta_{Gcc}$ | NA | -195.49 |
| 4 | 1.55 [1.23 - 1.97] | $\beta_{Lcc}$ | $\beta_{Lcc}*\theta$ | $\beta_{Lcc}*\theta$ | 1.46 [1.40 - 1.51] | $\beta_{Gcc}$ | $\beta_{Gcc}*\theta$ | $\beta_{Gcc}*\theta$ | 0.56 [0.43 - 0.73] | -195.40 |
| 5 | 1.57 [0.96 - 2.57] | $\beta_{Lcc}*\theta$ | $\beta_{Lcc}*\theta$ | $\beta_{Lcc}$ | 1.26 [1.08 - 1.49] | $\beta_{Gcc}*\theta$ | $\beta_{Gcc}*\theta$ | $\beta_{Gcc}$ | 0.79 [0.64 - 0.98] | -194.99 |
| 6 | 1.55 [1.47 - 1.64] | $\beta_{Lcc}$ | 0.87 [0.80 - 0.94] | $\beta_{Laa}$ | 1.16 [1.11 - 1.22] | $\beta_{Gcc}$ | 1.49 [1.24 - 1.80] | $\beta_{Gaa}$ | NA | -195.40 |
| 7 | 1.62 [0.81 - 3.24] | $\beta_{Laa}$ | 1.21 [1.09 - 1.34] | $\beta_{Lcc}$ | 1.23 [0.72 - 2.10] | $\beta_{Gaa}$ | 1.03 [0.85 - 1.25] | $\beta_{Gcc}$ | NA | -194.99 |
| 8 | 1.55 [0.84 - 2.88] | 1.47 [0.75 - 2.90] | 0.56 [0.02 - 16.16] | $\beta_{Lca}$ | 1.24 [1.22 - 1.27] | 1.41 [1.18 - 1.69] | 0.04 [0.00 – 1x10^25^] | $\beta_{Gca}$ | NA | -194.79 |
| 9 | 0.98 [0.59 - 1.64] | 1.13 [0.57 - 2.23] | 0.00 [0.00 – 7x10^215^ ] | 6.07 [2.36 - 15.59] | 0.00 [0.00 – 1x10^234^] | 1.77 [1.36 - 2.29] | 0.07 [0.00 – 1x10^3^] | 2.88 [1.01 - 8.24] | NA | -194.70 |

| 1. **Maindi village, Tanzania** | | | |  |  |  |  |  |  |  |
| --- | --- | --- | --- | --- | --- | --- | --- | --- | --- | --- |
| **Model number** | $\beta_{Lcc}$ | $\beta_{Lca}$ | $\beta_{Laa}$ | $\beta_{Lac}$ | $\beta_{Gcc}$ | $\beta_{Gca}$ | $\beta_{Gaa}$ | $\beta_{Gac}$ | $\theta$ | $\ln L$ |
| 1 | NA | NA | NA | NA | 2.36 [2.25 - 2.50] | $\beta_{Gcc}$ | $\beta_{Gcc}$ | $\beta_{Gcc}$ | NA | -375.37 |
| 2 | 1.97 [1.49 - 2.62] | $\beta_{Lcc}$ | $\beta_{Lcc}$ | $\beta_{Lcc}$ | 0.87 [0.62 - 1.22] | $\beta_{Gcc}$ | $\beta_{Gcc}$ | $\beta_{Gcc}$ | NA | -355.08 |
| 3 | 2.93 [2.18 - 3.95] | $\beta_{Lcc}$ | $\beta_{Lcc}$ | $\beta_{Lcc}$ | 1.37 [0.99 -1.90] | $\beta_{Gcc}$ | $\beta_{Gcc}$ | $\beta_{Gcc}$ | NA | -345.43 |
| 4 | 4.45 [3.21 - 6.16] | $\beta_{Lcc}$ | $\beta_{Lcc}*\theta$ | $\beta_{Lcc}*\theta$ | 2.66 [2.09 - 3.39] | $\beta_{Gcc}$ | $\beta_{Gcc}*\theta$ | $\beta_{Gcc}*\theta$ | 0.00001 [0.00 - ∞] | -343.33 |
| 5 | 2.59 [1.29 - 5.20] | $\beta_{Lcc}*\theta$ | $\beta_{Lcc}*\theta$ | $\beta_{Lcc}$ | 1.22 [0.08 - 1.84] | $\beta_{Gcc}*\theta$ | $\beta_{Gcc}*\theta$ | $\beta_{Gcc}$ | 1.26 [1.14 - 1.40] | -344.32 |
| 6 | 4.45 [3.19 - 6.19] | $\beta_{Lcc}$ | 0.01 [0.00 – 2x10^34^] | $\beta_{Laa}$ | 0.49 [0.00 – 2x10^11^] | $\beta_{Gcc}$ | 3.87 [0.01 - 1544] | $\beta_{Gaa}$ | NA | -343.33 |
| 7 | 3.83 [2.40 - 6.08] | $\beta_{Laa}$ | 2.49 [1.27 - 4.90] | $\beta_{Lcc}$ | 0.49 [0.02 - 1.58] | $\beta_{Gaa}$ | 2.06 [1.04 - 4.07] | $\beta_{Gcc}$ | NA | -342.94 |
| 8 | 3.18 [1.55 - 6.53] | 4.27 [2.77 -6.60 ] | 0 [0.00 – 9x10^29^] | $\beta_{Lca}$ | 0.81 [0.01 - 73.40] | 0.08 [0.00 – 9x10^28^] | 5.69 [1.19 - 27.25] | $\beta_{Gca}$ | NA | -340.88 |
| 9 | 2.79 [1.05 - 7.45] | 3.83 [2.01 - 7.30] | 0 [0.00 – 2x10^22^] | 6.08 [1.96 - 18.85] | 0.02 [0.00 – 5x10^53^] | 0.34 [0.00 – 7x10^14^] | 5.63 [0.20 - 160.62] | 0.44 [0.00 - 15195.13] | NA | -340.73 |
