# Supplementary material for: Targeting Antibiotics to Households for Trachoma Control
Source: PLoS Negl Trop Dis. 2010 Nov 2;4(11):e862. doi: 10.1371/journal.pntd.0000862 (PMC2970531; doi:10.1371/journal.pntd.0000862)
Supplement: Text S1 — Extra information of the methods: Model of Ocular Chlamydia transmission, Parameter Estimation, Model selection, Stochastic simulation and cost-effectiveness analysis. (0.42 MB DOC) [file pntd.0000862.s009.doc]

### Supporting Information Text

### Blake et al.

### This document describes the mathematical model of ocular *Chlamydia* transmission, the procedures for parameter estimation and model selection, the stochastic simulation model, and the details for the cost-effectiveness analysis.

## Model of Ocular Chlamydia Transmission

The model is an extension of our previous SIS household model [1]. The population is structured into households and to two age classes: ‘children’ (<10 years old), $c$, and ‘adults’ (≥10 years old), $a$. In each household there are $m_{c}$children and $m_{a}$adults; $j_{c}$infected children and $j_{a}$infected adults; and $\left(m_{c}-j_{c}\right)$susceptible children and $\left(m_{a}-j_{a}\right)$susceptible adults.

We assume frequency-dependent transmission of infection, in agreement with the results with our earlier study [1] , such that the hazard of infection is proportional to the fraction of contacts that are infectious (rather than their number). In this case, a susceptible individual of age class $y$is infected by a member of their own household (local transmission) of age class $x$ at a rate $\frac{\beta_{Lxy}j_{x}(t)}{\left(m_{x}-1\varepsilon_{xy}\right)}$ or by a member of their community (global transmission) of age class $x$at a rate$\beta_{Gxy}\nu_{x}$. The subscripts $x$ and $y$can either equal $c$ or $a$, and when$x=y$, $\varepsilon=1$and when$x\neq y$, $\varepsilon=0$. $\beta_{Lxy}$is the local transmission coefficient and $\beta_{Gxy}$ is the global transmission coefficient. Function $\nu_{x}(t)$is the global prevalence of infection in the population of age class $x$ as a function of time (*t*). There are therefore eight potential transmission parameters ($\beta_{Lcc}$, $\beta_{Lca}$, $\beta_{Laa}$, $\beta_{Lac}$, $\beta_{Gcc}$, $\beta_{Gca}$, $\beta_{Gaa}$and$\beta_{Gac}$) to estimate. Therefore a susceptible child in a household of size $m_{c}+m_{a}$ is infected at a rate $\lambda_{c}$, where $\lambda_{c}=\frac{\beta_{Lcc}j_{c}}{m_{c}-1}+\frac{\beta_{Lac}j_{a}}{m_{a}}+\beta_{Gcc}\nu_{c}+\beta_{Gac}\nu_{a}$. A susceptible adult in a household of size $m_{c}+m_{a}$is infected at a rate$\lambda_{a}$, where$\lambda_{a}=\frac{\beta_{Laa}j_{a}}{m_{a}-1}+\frac{\beta_{Lca}j_{c}}{m_{c}}+\beta_{Gaa}\nu_{a}+\beta_{Gca}\nu_{c}$. Children recover from infection at a rate $\gamma_{c}$and adults at a rate $\gamma_{a}$. Depending on assumptions there are a total of nine possible models that are nested in the most general model (described in Table S2). In model 4 adults are assumed to be *θ* times as infectious as children and in model 5 adults are assumed to be *θ* times as susceptible as children (where *θ* lies between 0 and *∞*). In those two models, the contact rate differs for household and community transmission but does not differ by age class.

The transmission dynamics within each household are described by a multi-state Markov model. The number of infected adults $j_{a}$ and children$j_{c}$, together with the number of children, $m_{c}$, and adults, $m_{a}$, in a household determine the state of the system at time $t$. For convenience we index this state by the function $s\left(t\right)=j_{a}\left(t\right)\left(m_{c}+1\right)+1+j_{c}\left(t\right)$. The total number of possible states a household can be in is given by $\left(m_{c}+1\right)\left(m_{a}+1\right)$.

The Markov transition intensities $q_{rs}\left(t\right)$ are determined by the instantaneous rate of infection among children $\left(m_{c}-j_{c}\right)\lambda_{c}$ (when $s=r+1$) and adults$\left(m_{a}-j_{a}\right)\lambda_{a}$ (when $s=r+m_{c}+1$), and the rate of recovery of children $j_{c}\gamma_{c}$ (when $s=r-1$) and adults$j_{a}\gamma_{a}$ (when $s=r-m_{c}-1$).

Collectively, the transition intensities form a matrix $\mathbf{Q}\left(m_{c},m_{a}\right)$ whose rows sum to zero so that $q_{rr}=-\sum_{s\neq r} q_{rs}$. The remaining matrix elements are zero. This matrix is completely determined by ten parameters: the eight transmission parameters and the two recovery rate parameters ($\beta_{Lcc}$,$\beta_{Lca}$, $\beta_{Laa}$, $\beta_{Lac}$, $\beta_{Gcc}$, $\beta_{Gca}$, $\beta_{Gaa}$, $\beta_{Gac}$,$\gamma_{a}$and $\gamma_{c}$). The matrix defining the transition probability of a household (of size $m_{c}+m_{a}$) being in state $s$ at a time $t+u$in the future, given the state at time $u$is $r$, is given by:

$\mathbf{P}\left(t,m_{c},m_{a}\right)=Exp\left(t\mathbf{Q}\right)$,

where $Exp$ is the matrix exponential function. The probability of a household being in state $s$ at endemic equilibrium, $z_{s}^{\left(m_{c},m_{a},j_{c},j_{a}\right)}$is determined by the corresponding diagonal entry of $\mathbf{P}\left(t,m_{c},m_{a}\right)$ as$t\to\infty$.

It was not possible to solve the endemic equilibrium for this model analytically but numerical methods can be used to find $\mathbf{P}\left(t,m_{c},m_{a}\right)$ from $\mathbf{Q}\left(m_{c},m_{a}\right)$as $t\to\infty$. time step, $\delta t$, $\to0$, the diagonal entries of $\mathbf{P}$,$p_{rr}\left(t\right)$,can be approximated to be $1+q_{rr}\delta t$and the other entries $p_{r\neq s}\left(t\right)\approx q_{rs}\delta t$. At endemic equilibrium, $z_{s}^{\left(m_{c},m_{a},j_{c},j_{a}\right)}$corresponds to the normalised left eigenvector, with an eigenvalue of 1, of the approximated transition probability time matrix $\mathbf{P}\left(t,m_{c},m_{a}\right)$.

### *Parameter Estimation*

Maximum likelihood was used to estimate model parameters from the cross-sectional, pre-intervention data on the prevalence of infection, assuming that infection was at endemic equilibrium. The likelihood,$L$, of observing a household of size $m_{c}+m_{a}$, in state $s$at equilibrium for a given set of model parameters is$L=z_{s}^{\left(m_{c},m_{a},j_{c},j_{a}\right)}$. The total log likelihood is the summation of $\ln L$ over all households.

A small number of children and adults were not tested for infection in each community. As described previously [1] if there were $\sigma_{x}$members of a household tested for infection, of age category $x$, then there are $\left(m_{x}-\sigma_{x}\right)$ who were not tested but could have contributed to transmission. Assuming these individuals do not differ from tested individuals with respect to their susceptibility to infection and infectiousness, the probability that $h_{x}$individuals were found positive given that $j_{x}$members were actually infected is described by a hypergeometric distribution [2],

$$P\left(H_{x}=h_{x}|j_{x},\sigma_{x},m_{x}\right)=\frac{\left(\begin{matrix} j_{x} \\ h_{x} \end{matrix}\right)\left(\begin{matrix} m_{x}-j_{x} \\ \sigma_{x}-h_{x} \end{matrix}\right)}{\left(\begin{matrix} m_{x} \\ \sigma_{x} \end{matrix}\right)}$$

The probability that a household was in state $S$ is therefore,

$P\left(S=s|j_{c},j_{a},\sigma_{c},\sigma_{a},m_{c},m_{a}\right)=P\left(H_{c}=h_{c}|j_{c},\sigma_{c},m_{c}\right)P\left(H_{a}=h_{a}|j_{a},\sigma_{a},m_{a}\right)$.

The likelihood for a household is modified such that,

$L=\sum_{s=1}^{s=\left(m_{c}+1\right)\left(m_{a}+1\right)} \left[z_{s}^{\left(m_{c},m_{a},j_{c},j_{a}\right)}P\left(S=s|j_{c},j_{a},\sigma_{c},\sigma_{a},m_{c},m_{a}\right)\right]$.

The variance of each parameter estimate was obtained from the diagonal entries of the inverted Hessian matrix of the maximized log-likelihood [3]. Approximate confidence intervals were calculated assuming a normal distribution on the log-scale for the parameters.

To maximize the likelihood, the Nelder-Mead optimization method was used from the ‘optim’ R package. To ensure that a global maximum likelihood was found, optimization was restarted at different parameter values and robustness of the estimate was examined under both Nelder-Mead and simulated-annealing algorithms.

### *Model Selection*

Incorporating two age groups and household structure into the model of ocular chlamydial transmission described in *SI Text Model of Ocular Chlamydia Transmission* allows for up to eight transmission parameters to be estimated. However simpler models are special cases within this eight transmission parameter model. For each dataset, the Akaike Information Criterion (AIC) was used to identify the most parsimonious yet adequate model [4]. The AIC is given by $AIC=-2L+2k$, where $L$is the total log-likelihood and $k$is the number of parameters estimated. When comparing two models, a difference greater than 10 between the respective AICs is considered as empirical evidence in favor of the model with the lower AIC value [4].

Table S2 shows the AIC of each model for each community. The difference in AIC values between models 1 (homogeneous mixing of the population with no age structure) and 2 (household and community transmission, no age structure) is larger than 10 in all four settings in favor for model 2, and so there is support for a model with household and community transmission. There is also support for including age structure in addition to household structure when the model includes a shorter duration of infection for older individuals compared with younger individuals (model 3). The exception is the Upper Saloum District in The Gambia. In this case the difference in the AIC values is less than ten between models with or without explicit age structure.

The fit to the data (excluding Kahe Mpya sub-village in Tanzania) improves if differences in the household and community transmission rates depend on the age of the susceptible and infectious individuals (models 4–9). Only in Jali village, The Gambia, is the reduction in the AIC between models 5 and 3 large enough for there to be support for model 5 (adults are assumed to be less susceptible to infection than children). The parameter estimates for the different models are given in Table S6.

In all four datasets there was strong evidence for household and community transmission over a model with simple homogeneous mixing of a population. Three out of four communities had strong evidence for children having a longer duration of infection than adults, with the exception of the Upper Saloum District. This community had a very low prevalence of infection (7%) and therefore older individuals may not have had enough exposures to infection for a partially protective immune response that would reduce the duration of infection. Prevalence of infection has previously been shown not to decline with age in this population [5]. Adding a further level of complexity (assuming that individuals aged ≥ 10 years old were less susceptible to infection than those aged < 10) provided a better explanation of the data from Jali village. Including this and other levels of complexity such as different mixing patterns between the two age groups did not provide strong evidence for a better explanation of the data in the other communities. Larger studies would be required to estimate such phenomena.

## Stochastic Simulation Model

The stochastic simulation model was based on the model outlined in *SI Text Model of Ocular Chlamydia Transmission*. Each household, $i$, is divided into four state variables: susceptible children, $H_{i,1}$, infected children, $H_{i,2}$, susceptible adults, $H_{i,3}$, and infected adults, $H_{i,4}$. The number of new infections, $I_{i,y}$, and recoveries, $R_{i,y}$, from infection in both age groups were assumed to be binomially distributed (number of trials, probability), where $y$ is the age group index:

- - - $I_{i,c}\left(t\right)$~binomial($H_{i,1}\left(t\right)$,$\lambda_{c}\left(t\right)\delta t$) and $I_{i,a}$~binomial($H_{i,3}\left(t\right)$,$\lambda_{a}\left(t\right)\delta t$)
    - $R_{i,c}\left(t\right)$~binomial($H_{i,2}\left(t\right)$,$\gamma_{c}\delta t$) and $R_{i,a}\left(t\right)$~binomial($H_{i,4}\left(t\right)$,$\gamma_{a}\delta t$)
    - Therefore$H_{i,1}\left(t+dt\right)=H_{i,1}\left(t\right)+R_{i,c}\left(t\right)-I_{i,c}\left(t\right)$,$H_{i,2}\left(t+dt\right)=H_{i,2}\left(t\right)+I_{i,c}\left(t\right)-R_{i,c}\left(t\right)$, $H_{i,3}\left(t+dt\right)=H_{i,3}\left(t\right)+R_{i,a}\left(t\right)-I_{i,a}\left(t\right)$and $H_{i,4}\left(t+dt\right)=H_{i,4}\left(t\right)+I_{i,a}\left(t\right)-R_{i,a}\left(t\right)$

##### **Implementation of Treatment.** The model was run to equilibrium and at chosen time points (depending on the level of endemicity) treatment was implemented. Four treatment strategies were investigated using the stochastic simulation model:

a) Mass treatment in which the aim is to treat everyone in the community but a certain proportion, 1-$\eta$, of individuals is missed;

b) Targeted treatment of households with one or more members presenting with active disease in a household but a proportion of individuals (1-$\eta$) is missed;

c) Targeted treatment of households with one or more member presenting with active disease and all members within such household are treated;

d) Mass treatment of only children aged <10 years, assuming a certain proportion, 1-$\eta$, is missed.

The number of individuals treated in each state variable $H_{i,y}^{treated}$in each strategy were assumed to be distributed as:

a) $H_{i,y}^{treated}$~ binomial ($H_{i,y}\left(t\right)$,$\eta$),

b) $H_{i,y}^{treated}$~ binomial ($H_{i,y}\left(t\right)$,),

c) $H_{i,y}^{treated}$~ binomial ($H_{i,y}\left(t\right)$,) and

d) $H_{i,y}^{treated}$~ binomial ($H_{i,c}\left(t\right)$,$\eta$).

Parameter $\eta$ (therapeutic coverage) was set to equal 0.80. Parameter indicates whether a household has one or more individuals with active disease (which is referred to as a ‘diseased’ household); =1 if the household is ‘diseased’ and =0 if the household has no diseased individuals. The probability that a household is ‘diseased’$\omega_{i}\left(j_{i}\left(t\right)\right)$, as a function of the number of infected individuals at time $t$, was calculated from the data for each of the four communities (Figure S3). If the number of infected individuals in a household was greater than three, the probability that the household was diseased was assumed to be one. At each treatment time was determined for each household: ~binomial (1,$\omega\left(j\left(t\right)\right)$.

Azithromycin was assumed to be 95% efficacious in clearing the infection [6]. The number of successfully treated individuals was binomially distributed with a probability of 0.95 and was sampled from the number of infected individuals selected for treatment. Successfully treated individuals at the time of treatment were transferred from the respective age-class infected household state variable to the corresponding susceptible state variable.

##### **Household Heterogeneity.**

##### Accounting for variation in transmission parameters among households (for example because of differences in hygiene) was explored. Each household was assigned a relative transmission potential, $\pi_{i}$, which was sampled from the negative binomial distribution (to reflect overdispersion in household hygiene) with a mean of 1 and a overdispersion parameter $\kappa$ . The child and adult forces of infection for each household were modified to be $\pi_{i}\lambda_{c,i}$and $\pi_{i}\lambda_{a,i}$. Parameter$\kappa$was varied to explore the effect of different levels of overdispersion.

## Cost-Effectiveness Analysis

#### Calculation of mean number of tablets received by each age category.

##### **Data.** From 11th April to 26th June 2000, a village-wide trachoma prevalence survey was undertaken in Kahe village, Rombo district, Tanzania, as a prelude to the longitudinal studies of Kahe Mpya sub-village described elsewhere [7]. Every non-pregnant resident of Kahe Mpya (the "cohort" sub-village) was offered a single oral dose of approximately 20mg/kg azithromycin (to a maximum of 1g) after baseline examination and swabbing in that longitudinal study. The field team then returned to the six other sub-villages of Kahe to administer antibiotic treatment there. The same criteria and dosage schedule was employed for azithromycin treatment. An initial round of balozi-to-balozi (balozis are households within the same geographical zone that share the same balozi leader) visits was conducted, after giving notice to residents that antibiotic would be available at their balozi leader’s house on a specified day. Several mop-up exercises were then undertaken in an attempt to make sure that no-one who wanted treatment missed out. Azithromycin treatment was directly observed in every instance.

In accordance with national treatment guidelines, in both the cohort and non-cohort sub-villages, women who said they were pregnant and children under the age of twelve months were offered two tubes of 1% tetracycline eye ointment, in place of azithromycin. Including individuals in both the "cohort" and "non-cohort" sub-villages and treatment with either (oral) azithromycin or (topical) tetracycline, the total number of individuals treated in Kahe village was 5,016 against a denominator of 5,853, giving an overall treatment coverage of 85.7%.

##### **Calculation.** The data were split into the two age classes used in this work and the mean number of azithromycin tablets received in each age class was calculated.

#### Lower and Upper Bounds for Cost-Effectiveness Ratios.

The cost-effectiveness intervals were defined using a conservative approach, such that lower bounds were estimated by calculating the incremental cost-effectiveness ratio for each simulation using the lower costs estimates and taking the lower quartile of these ratios. The upper bounds were estimated by calculating the incremental cost-effectiveness ratio for each simulation using the upper costs estimates and taking the upper quartile of these ratios.

## References

1. Blake IM, Burton MJ, Bailey RL, Solomon AW, West S, et al. (2009) Estimating household and community transmission of ocular Chlamydia trachomatis. PLoS Negl Trop Dis 3: e401.

2. Johnson NL, Kotz S, Kemp AW (1992) Hypergeometric Distributions. Univariate Discrete Distributions. New Jersey: Wiley and Sons.

3. Bolker BM (2008) Likelihood and All That. Ecological Models and Data in R. 1st ed. Princeton: Princeton University Press. pp. 169 - 220.

4. Burnham K, Anderson D (2004) Model Selection and Multi-model Inference: A Practical Information-theoretic Approach. New York: Springer. 496 p.

5. Burton MJ, Holland MJ, Faal N, Aryee EA, Alexander ND, et al. (2003) Which members of a community need antibiotics to control trachoma? Conjunctival Chlamydia trachomatis infection load in Gambian villages. Invest Ophthalmol Vis Sci 44: 4215-4222.

6. Bailey RL, Arullendran P, Whittle HC, Mabey DC (1993) Randomised controlled trial of single-dose azithromycin in treatment of trachoma. Lancet 342: 453-456.

7. Solomon AW, Holland MJ, Alexander ND, Massae PA, Aguirre A, et al. (2004) Mass treatment with single-dose azithromycin for trachoma. N Engl J Med 351: 1962-1971.
